# Dental Adhesion Protocol: A Clinically Oriented Literature Review with Practical Guidelines

**DOI:** 10.3390/dj14030189

**Published:** 2026-03-23

**Authors:** Almerinda Agrelli, Mateus do Vale Voigt, Victor G. R. Clavijo, Lucas Coêlho Bernardo-Menezes, Ricardo Malise, Adilson dos Santos Torreão, Dione Maria Viana do Vale, Clarice Neuenschwander Lins de Morais

**Affiliations:** 1 Laboratory of Virology and Experimental Therapy (LaViTE), Aggeu Magalhães Institute, Oswaldo Cruz Foundation, Recife 50740-465, PE, Brazil; lucascoelhobernardo.lb@gmail.com (L.C.B.-M.);; 2 Private Practice, Dentistry Center Adilson Torreão, Recife 51110-000, PE, Brazil; 3 Advanced Program in Operative and Adhesive Dentistry, University of Southern California, Los Angeles, CA 90089, USA; 4 Facial Defects Care Center, Prof. Fernando Figueira Integral Medicine Institute (IMIP), Recife 50070-550, PE, Brazil

**Keywords:** absolute isolation, adhesive systems, dental adhesives, rubber dam isolation, self-etch, total etch

## Abstract

**Background:** Dental adhesive materials are important for achieving adequate adhesion results; however, they are not the only factor contributing to final bond strength, as improper operatory field isolation and contamination also significantly influence clinical outcomes. **Objectives:** This narrative review aims to provide a clinical perspective, supported by evidence-based arguments, to identify clinical procedures for optimizing adhesive protocols, including the execution of absolute isolation with a rubber dam, appropriate cleaning and preparation of the dental substrate, and protocols applicable to total-etch and self-etch techniques. **Methods:** The literature included in this review was selected through a structured search in PubMed, Scopus, and Web of Science, prioritizing systematic reviews, meta-analyses, long-term clinical studies, and foundational experimental investigations related to adhesive systems and substrate management. **Results:** A well-established clinical protocol that integrates proper adhesive selection, contamination-free operative field control, and adequate substrate preparation is essential for achieving predictable outcomes in adhesive dentistry. **Conclusions:** Although simplified adhesive systems offer acceptable bond strength results, established techniques continue to demonstrate consistent reliability, contributing to restorative longevity.

## 1. Introduction

The advancement in the development of simplified adhesive systems has introduced numerous advantages, especially in reducing the clinical operational time. However, this simplification resulted in an increased number of techniques to perform dental adhesion, often leading to confusion on what type of adhesive is being used and what is the correct protocol for that specific adhesive system. The extensive range of available adhesive brands further complicates the selection of the correct protocol to be used [1].

Various studies have sought to simplify the use of adhesive systems by standardizing protocols for each established technique, aiming to reduce procedural variability and improve clinical results [1,2,3,4]. These studies assist clinicians in selecting the most effective adhesive materials and protocols for each clinical situation. The establishment of these protocols reduces the probability of clinical errors and increases efficiency in adhesive procedures within restorative dentistry.

While the selection of the adhesive system is an important factor in achieving acceptable bond strength results, it is not the only factor that influences the clinical outcome. For instance, effectively isolating the operative field is essential to prevent contamination and reduce the overall humidity that may hinder the effectiveness of the adhesive interface [2].

The longevity of the adhesive interface is not solely determined by the selection of the adhesive system but also by the execution of the correct protocol for each type of adhesive system. A thorough approach that incorporates material selection, procedural standardization, and compliance with evidence-based protocols is critical for achieving consistent and reliable clinical outcomes [4].

The clinician should consider other equally important factors, including isolation of the operatory field, surface cleaning, and substrate treatment. An approach that integrates these elements is fundamental for achieving long-term clinical success in adhesive dentistry.

Therefore, this article aims to analyze adhesive systems, clinical protocols, and techniques for optimizing their performance. It is divided into three sections: (1) rubber dam isolation and materials; (2) preparation of the dental substrate for optimal bonding; and (3) adhesive techniques and materials.

The first section will address the importance of rubber dam isolation for a good adhesion outcome and how to properly use the rubber dam materials like the sheets and clamps, among others. As an article with a focus on the clinical perspective, insights about how to make proper punching and how to correctly position the clamps to achieve the best proper sealing with the rubber dam will be also addressed. In the second section, this article will examine the preparation of the substrate necessary to optimize the adhesive process. Key topics will include cleaning protocols designed to eliminate debris and contaminants that may compromise adhesion and how to simplify the cleaning and preparation in complex substrates with dentin exposure. The final section will concentrate on the selection and protocols of adhesive systems, especially the ones considered references of dental adhesion. Additionally, this section will provide the best protocols for standard adhesive systems for total-etch and self-etch techniques. The performance of adhesive systems in various restorative contexts, including direct composite restorations and indirect restorations such as onlays, inlays, ceramic veneers, and crowns, will also be analyzed to give a clinical view of different approaches.

By systematically addressing all essential steps, from isolation and substrate preparation to adhesive application, this article aims to equip clinicians with the essential knowledge and techniques needed to achieve consistent and predictable results in adhesive dentistry, ultimately enhancing long-term treatment success. In this regard, the goal of the current narrative review is to compile clinically significant data regarding adhesive procedures. Three interconnected elements that affect adhesive performance—rubber dam isolation and contamination control, substrate preparation and surface conditioning, and adhesive systems and application procedures—form the framework of the conversation. These elements were chosen because they have been consistently linked in the literature to restoration survival and bond durability.

## 2. Rubber Dam Isolation

**Figure 1:**
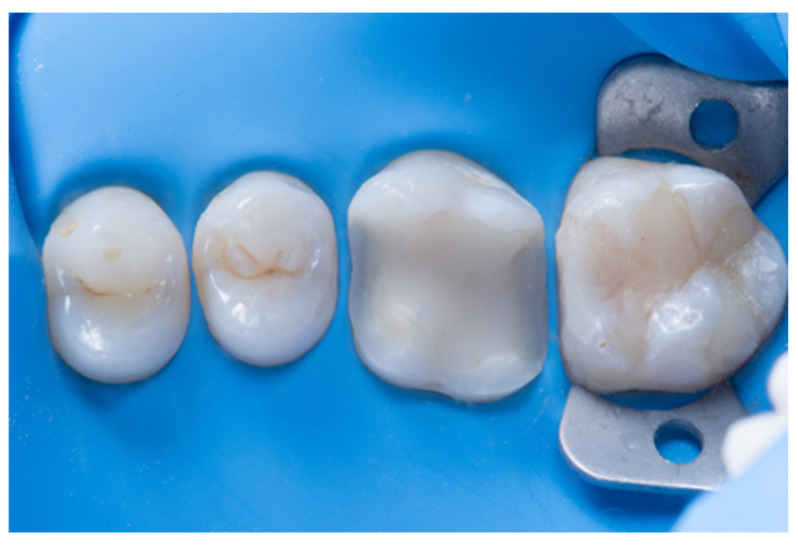
The optimal condition for adhesive procedures: low humidity, no saliva or blood, and good visualization of the operative site.

Effective dental adhesion requires a clean, dry, and contamination-free operative environment (Figure 1). Laboratory and clinical studies consistently demonstrate that increased relative humidity and biological contaminants—such as saliva, blood, biofilm, and hemostatic agents—negatively affect bond strength and interfacial stability [5,6,7]. Recent reviews further suggest that moisture-related degradation accelerates hydrolytic breakdown of resin–dentin interfaces, particularly in simplified adhesive systems [8].

Although direct clinical trials comparing adhesive outcomes with and without rubber dam isolation remain limited, converging evidence indicates that environmental control is a prerequisite for durable adhesion. Rubber dam isolation remains the most reliable method for achieving stable humidity control, preventing salivary contamination, improving visualization, and enhancing operator precision [9,10].

Recent clinical perspective articles have reinforced that inadequate training and unfamiliarity with rubber dam techniques—not inherent limitations of the method—represent the primary barriers to its routine use. Improper punching patterns, inappropriate clamp selection, and insufficient sealing can lead to fluid leakage and patient discomfort. When correctly executed, however, rubber dam isolation consistently improves procedural efficiency and adhesive reliability [6,7,8,9,10,11,12,13,14,15,16].

A lack of proficiency in rubber dam isolation may lead to complications such as periodontal injury, tearing of the rubber sheet, patient discomfort due to improper clamp placement, and the potential risk of fracturing a structurally compromised tooth. Furthermore, inadequate isolation may result in fluid leakage due to an improper seal, which can stem from incorrect punching of the rubber sheet or the selection of an inappropriate rubber sheet [6].

To properly use the rubber dam isolation, some guides should be followed:

2.1 Selection of the rubber sheet;
2.2 Selection of the area of isolation;
2.3 Proper punching;
2.4 Proper sealing;
2.5 Selection of the clamps.

### 2.1. Selection of the Rubber Sheet

To understand how to select the appropriate rubber dam sheet for each case, it is essential to first recognize the different types of sheets and their impact on the final isolation. Rubber dam sheets can be distinguished by their thickness, size, and composition. While thicker sheets offer better protection for oral tissues, provide more effective containment of intra-oral fluids, and are more resistant to tearing, thinner sheets are easier to pass through contact points and more comfortable for the patient. However, thinner sheets are more prone to tearing and often fail to adequately contain intra-oral fluids. The primary drawbacks of thicker sheets include the difficulty in passing them through proximal contacts and the tension they exert on the most posterior clamps, which may lead to their displacement [6].

**Figure 2:**
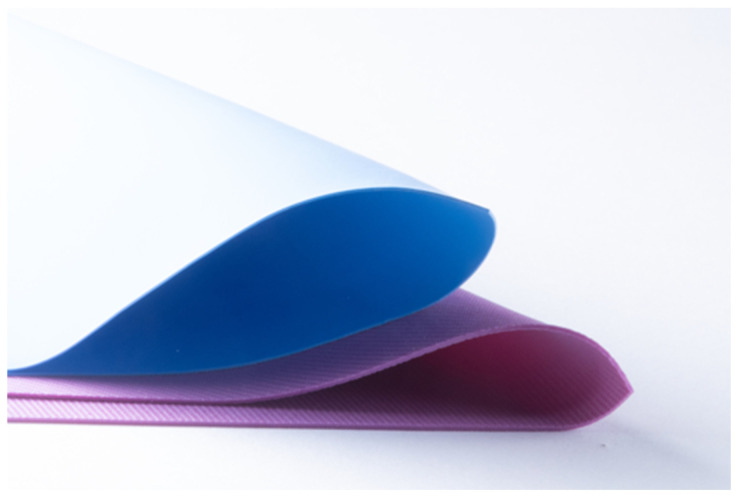
Example of two rubber dam sheets, Nic Tone (blue) and Flexi Dam (purple).

There are two sizes of rubber dam sheets: 5 × 5 inches and 6 × 6 inches. The 5 × 5-inch sheet is typically used for children, while the 6 × 6-inch sheet is recommended for adults. The 6 × 6-inch sheet is generally preferred, as it can be paired with a smaller frame, providing greater flexibility for adjusting the sheet’s tension [7]. The composition of the sheet is also an important factor in case-by-case selection. Rubber dam sheets are available in latex and non-latex options, making it essential to consider patients with latex allergies. The primary difference in composition lies in its effect on the sheet’s elasticity. More elastic sheets provide less tissue retraction and reduced containment of fluids, whereas less elastic sheets offer greater tissue retraction but can be more challenging to manipulate. The non-latex options are typically more flexible (e.g., Flexi Dam, Coltene, Altstätten, Switzerland). However, for both latex and non-latex rubber dam sheets, elasticity may vary depending on the manufacturer. Most cases can be properly isolated with a medium-gauge rubber dam sheet (e.g., Nic tone, MDC Dental, Ciudad de México, Mexico) (Figure 2) [8].

### 2.2. Selection of the Area of Isolation

To determine the appropriate area for isolation, several factors must be considered, including which teeth will be restored (anterior or posterior), the number of teeth involved, and whether the restoration extends to the proximal surfaces. For anterior teeth, a general guideline is to extend the isolation of at least two teeth beyond the most posterior tooth being restored. For posterior teeth, the isolation should extend at least one tooth posteriorly and two teeth anteriorly to the tooth being treated. If only a single tooth is being restored and the restoration does not involve the proximal surfaces, isolation can be achieved with a single punching for the tooth undergoing treatment.

### 2.3. Proper Punching

**Figure 3:**
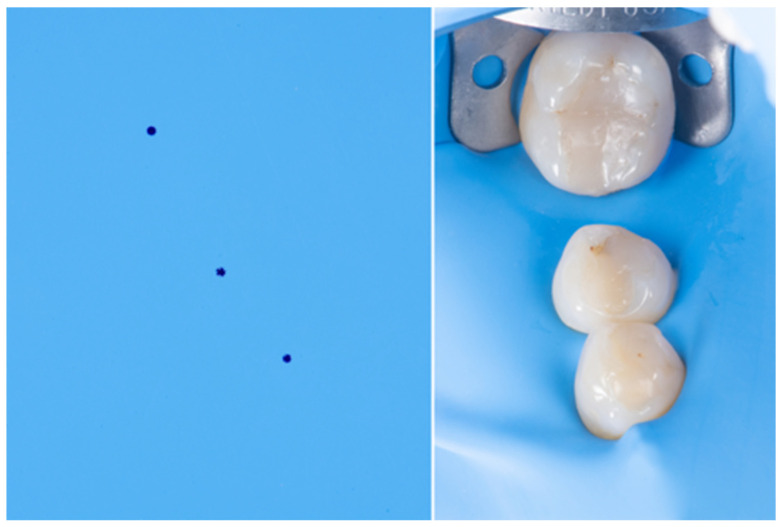
The image on the left shows an increased distance between the molar and the pre-molar markings due to the presence of diastema. The image on the right is post-rubber dam isolation; notice the proper settlement of the sheet in the diastem area.

It is important to emphasize that the portion of the rubber dam covering the papillary region remains unchanged. Therefore, the punching pattern should account for the interproximal distances to ensure proper spacing between the holes. As a general guideline, punchings should be placed 5 mm apart. For molars, the punchings should be 7 mm apart, considering that the hole created by the puncher already occupies approximately 1.5 mm per punching. In patients with diastemas, the punching spacing should be increased by at least 1.5 mm in addition to the standard 5 m for most teeth or 7 mm for molars to ensure proper adaptation of the rubber dam. There are punching guides available on the market that can assist with the initial marking of the punchings. If any adjustments are required, such as in cases of diastemas that requires at least 1 mm extra of distance between markings, they should be made after the initial marking (Figure 3).

### 2.4. Proper Sealing

A proper seal of the teeth by the rubber dam sheet is paramount for effective isolation. Inadequate sealing may allow fluids to bypass the barrier, leading to contamination of the tooth being treated. Improper sealing can result from the tearing of the sheet, failure of the sheet to pass through the interproximal contact, or interference from the clamp, preventing the sheet from properly adapting to the tooth [5,6].

For proper sealing, several key aspects must be considered. The insertion of the rubber dam through the contact points should be performed gently to prevent damage to the sheet or gingival tissues. One technique involves mimicking the action of dental floss and applying buccopalatal tension while carefully guiding the rubber dam through the contact point. Alternatively, waxed dental floss (preferably a wider type) can be used to progressively guide the rubber dam through the contact. This process begins by applying pressure on the occlusal surface of one tooth until one end of the sheet passes through the contact. The same maneuver is then repeated on the occlusal surface of the adjacent tooth, ensuring that the rubber dam is properly positioned around the interdental papillae for optimal isolation [16].

To achieve an effective seal of the rubber dam around the tooth with the primary clamp (typically the most posterior tooth), additional steps are required if it is not the last tooth in the patient’s arch. In such cases, dental floss should be carefully passed around the distal side of the clamp, ensuring it secures the rubber dam in place. The floss is then guided through the proximal contact, creating an effective seal on the distal aspect of the tooth holding the main clamp [17].

The rubber dam may often become trapped on the primary clamp, preventing proper contact between the sheet and the tooth. To avoid this issue, the clinician should secure the rubber dam in place while gradually releasing one side of the clamp at a time using clamp forceps. This controlled adjustment allows the rubber dam to adapt correctly around the tooth, ensuring an effective seal and optimal isolation [5,6,16].

### 2.5. Selection of the Clamps

To select the appropriate clamp for each case, it is essential to first understand the function of each type. Clamps can be categorized as retentive clamps (primary clamps) and retraction clamps (auxiliary clamps). Primary clamps are placed on the most posterior tooth to secure the rubber dam in position, ensuring a stable and unobstructed operative field for the clinician. Auxiliary clamps, on the other hand, are used to retract the gingival tissue, providing better exposure to the area to be restored [13].

**Figure 4:**
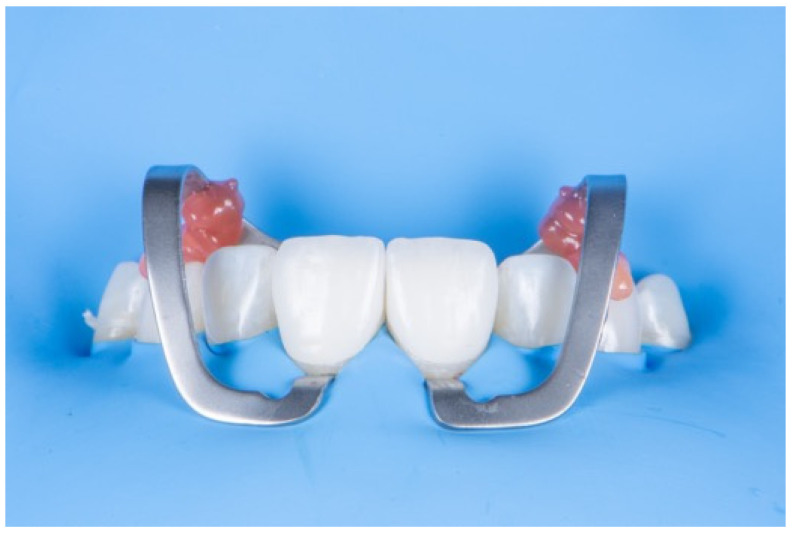
An example of auxiliary clamps with flowable composite stabilization.

To ensure the stability of the clamp, it should function like a chair, with four contact points ideally distributed as far apart as possible. If the clamp has only three or fewer contact points or if there is an area of continuous contact on one or both sides, it will become unstable, allowing the rubber dam to shift or even dislodge the clamp from the tooth. Auxiliary clamps are typically thinner to enable tissue retraction with minimal to no damage to the gingiva. However, due to their reduced thickness, they offer less stability and may require additional reinforcement, often achieved by applying flowable composite for stabilization (Figure 4) [13].

## 3. Substrate Preparation

Following isolation, substrate preparation represents a critical step in optimizing adhesive performance. The contemporary literature reinforces that enamel and dentin must be approached as distinct substrates, each requiring tailored surface treatment. While enamel adhesion remains highly predictable following phosphoric acid etching, dentin bonding continues to present challenges due to its organic composition, intrinsic moisture, and tubular structure [4,18,19].

Recent studies highlight that residual contaminants and smear layer characteristics significantly influence resin infiltration and hybrid layer formation. Mechanical cleaning with pumice and water remains effective for superficial debris removal; however, its ability to uniformly treat complex dentin topography is limited [15]. Consequently, surface roughening using low-speed carbide burs has been advocated to enhance micromechanical retention, albeit at the cost of increased clinical time and technique sensitivity [17,20].

Air particle abrasion (APA) has gained renewed attention in the recent literature as a means of simultaneously cleaning and conditioning dental substrates. Narrative and systematic reviews indicate that APA—when performed with controlled particle size and pressure—can increase surface energy, improve wettability, and enhance adhesive penetration [19,21]. Nevertheless, heterogeneity among study protocols and equipment underscores the importance of cautious parameter selection and operator training.

**Figure 5:**
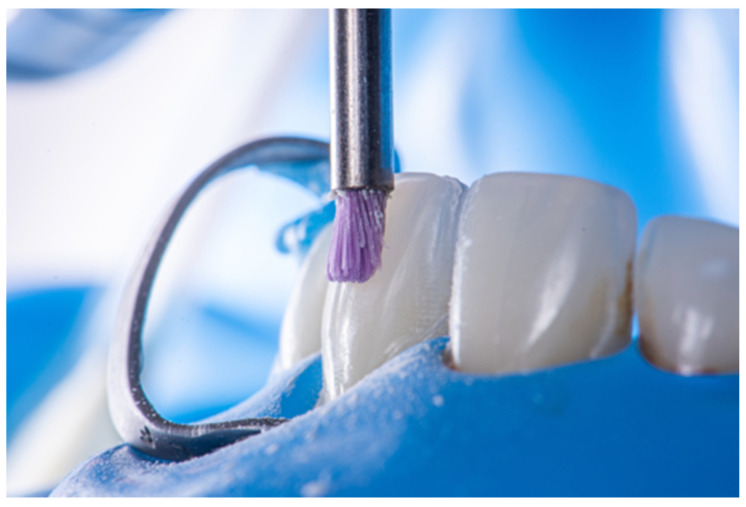
Mechanical cleaning with a fine tip brush.

**Figure 6:**
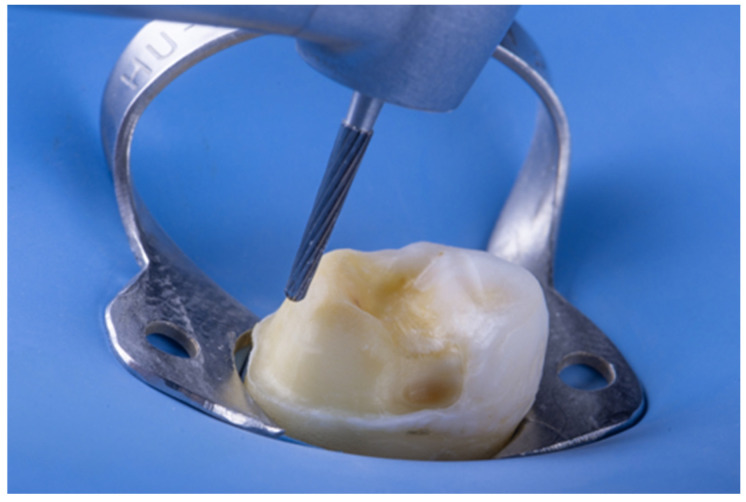
Dentin roughening with carbide bur.

Overall, current evidence [1,4] supports a substrate-driven approach, in which cleaning and roughening strategies are selected based on clinical accessibility, substrate exposure, and restorative indication. When combined with adequate isolation, optimized substrate preparation contributes significantly to the durability of the adhesive interface (Figure 5 and Figure 6).

**Figure 7:**
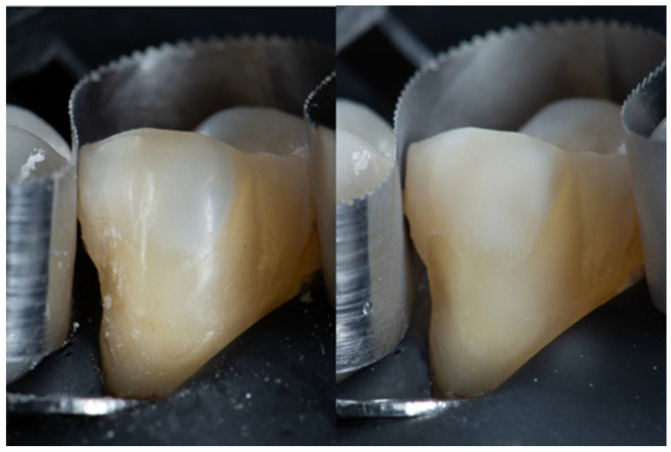
The left image shows a rubber dam isolated tooth with contaminated surface. The image on the right shows the clean surface after APA cleaning.

Air Particle Abrasion (APA) protocol is applicable for both enamel and dentin. It employs air abrasion with bioactive glass or alumina particles to simultaneously clean and roughen the substrate. To ensure optimal performance, the particle size should be less than 50 microns, applied at a pressure of 4 bar for approximately 5 s. This technique provides a more homogeneous surface treatment, facilitates access to difficult areas, and improves adhesive bonding by increasing surface energy and micromechanical retention (Figure 7) [19,21].

## 4. Adhesive Systems

Advances in adhesive dentistry have produced a wide spectrum of etch-and-rinse, self-etch, and universal adhesive systems. Despite these innovations, long-term data continue to favor multi-step strategies with separate conditioning and priming phases, particularly for dentin bonding [2,3,22]. Recent meta-analyses and consensus reports indicate that simplified all-in-one adhesives remain more susceptible to hydrolytic degradation and nanoleakage over time [16,23].

Universal adhesives have demonstrated versatility across multiple substrates; however, their clinical performance is strongly influenced by application mode, solvent evaporation, and enamel pre-treatment. Current evidence suggests that selective enamel etching enhances enamel bond strength when using universal systems, while dentin outcomes remain material dependent [24,25].

Importantly, the persistent classification of OptiBond FL and Clearfil SE Bond as reference systems reflects not only their chemical formulation but also the breadth and duration of supporting evidence, including laboratory studies, clinical trials, and long-term follow-up evaluations (4). In this context, the designation is justified as a reference benchmark grounded in cumulative scientific evidence and long-term validation rather than in technological novelty.

- The demineralization of the tooth structure: Since Buonocore [19], phosphoric acid etching of the tooth structure has been one of the foundations of dental adhesion. Acid etching of enamel demineralizes the structure, creating microretentions, where the dental bonding agent will interlock and form a hybrid layer when polymerized [26,27,28,29,30]. Acid etching performed on dentin results in partial demineralization, exposing the collagen fibril network by dissolving the mineral phase (hydroxyapatite) [28,31,32,33]. This process allows resin monomers to infiltrate the exposed collagen and form a hybrid layer when polymerized. With the advancement of adhesive systems, self-etch systems were introduced, in which the adhesive primer penetrates the dentin while simultaneously promoting demineralization. Although this technique is effective on dentin, the acid is not strong enough to create microretentions in enamel in the same way that phosphoric acid does.
- The infiltration of the dentin: Due to the structure of dentin and the presence of dentinal fluids, it is challenging for the “bond” of adhesive systems to penetrate dentin, as it is a hydrophobic material. For this reason, it is necessary for a hydrophilic material (the primer) to penetrate the demineralized dentin region and subsequently copolymerize with the adhesive bond, creating the hybrid layer and the micromechanical retention required for adequate bond strength. With self-etch systems, the infiltration of dentin occurs as the acidic primer demineralizes the dentin hydroxyapatite [34].
- The bonding agent: The bonding agent, or “bond,” in multi-bottle adhesive systems, mechanically interlocks with the demineralized enamel and dentin. Upon polymerization, it forms the hybrid layer, a bio-composite structure at the interface between demineralized dentin, enamel, and the bonding agent. As a structure primarily composed of methacrylate, the hybrid layer establishes a chemical bond with methacrylate-based materials such as resin composites, flowable composites, and resin cement [3].

The adhesion strategies for each case will have some variables, such as whether the substrate will be enamel or dentin, which cement will be chosen for cementation, etc. Initially, it is necessary to classify the materials available on the market according to the type of conditioning they promote/require and the influence of this conditioning on the dental substrate. In this context, adhesives can be classified into: (1) total-etch adhesives, which require prior conditioning with phosphoric acid before applying the adhesive system; (2) self-etch adhesives, which contain an acidic primer in their composition that acts as the phosphoric acid, and finally, (3) universal adhesives (multi-mode), which can be used with the total-etch technique (with phosphoric acid conditioning) or self-etch (without phosphoric acid conditioning) [3].

The total-etch strategy involves the use of 30–40% phosphoric acid in a separate step, applied to the dental substrate for 15 to 30 s and then rinsed off. The effect of this acid etching is different when applied to enamel and dentin. Acid etching on enamel promotes selective dissolution of enamel prisms, creating microporosities that will be infiltrated by the adhesive. It is now well established that the adhesive procedure with acid etching on enamel followed by adhesive application is the most reliable adhesion procedure, presenting high success rates. Conversely, dentin, being composed of 50% inorganic matrix, 30% organic substance, and 20% water, has some particularities. In this case, acid etching acts differently and requires greater care. When performed, acid etching on dentin promotes demineralization of peritubular dentin, exposing a network of collagen fibers that will later be infiltrated by the adhesive. However, this network must not remain dry or be air-dried after acid etching, as this can collapse the collagen fiber network and inhibit adhesive infiltration. Additionally, the contact time between the acid and dentin should not exceed 15 s, as there is a risk of collagen fiber network destruction. For this reason, acid etching on dentin is one of the most technique-sensitive adhesive techniques [35,36].

For the total-etch strategy, adhesives are available in two presentations: two-bottle adhesives (separate primer and bond—4th Generation) and single-bottle adhesives (primer + bond—5th Generation). In these systems, the primer is a hydrophilic monomer that penetrates the collagen fibers of dentin (which, under normal conditions, contain water), facilitating bond penetration since adhesive bonds are hydrophobic. In enamel, which has a high mineral composition and therefore low water content, the function of the primer as an adhesion facilitator is unnecessary. In the case of the two-bottle adhesive, the application control of the primer and bond is greater; however, due to the increased number of steps, the technique becomes more sensitive and prone to operator errors [37,38,39,40].

Self-etch adhesives were introduced to address the technique sensitivity of dentin conditioning. With advancements in acidic monomers such as MDP (10-methacryloyloxydecyl dihydrogen phosphate), 6th Generation adhesives, available in two bottles (acidic primer and bond), promote primer penetration into dentin while simultaneously demineralizing it, reducing or almost eliminating the risk of collagen fiber network collapse. Besides simplifying the dentin conditioning technique, the bond strength results are comparable to more recent adhesive systems. These adhesives can also be used with the selective enamel conditioning technique, where phosphoric acid is applied only to enamel, and then the primer is applied to dentin. The bond is then applied to the entire substrate, increasing adhesion strength in enamel. Care must be taken when using selective enamel conditioning with these adhesives because they depend on the smear layer for good adhesion to dentin, and acid runoff onto dentin can reduce its adhesion [40,41,42].

To further simplify the adhesive technique, 7th Generation adhesives, or self-etch adhesives available in a single bottle, were introduced. These adhesives combine the acidic primer and bond in one device, simplifying their use; however, they demonstrate lower bond strength compared to two-bottle systems, which still provide the best results [34].

Universal Adhesives: Universal adhesives (8th Generation), also known as multi-mode adhesives, contain components in a single bottle that allow for the total-etch or self-etch technique, with or without selective enamel conditioning. A combination of hydrophobic and hydrophilic components enables multiple adhesive actions on different substrates. The ability to be used with any technique (total etch or self-etch) eliminates the issue of self-etch adhesives not tolerating acid runoff onto dentin. Thus, if acid runoff occurs when using universal adhesives, no complications arise, minimizing operator errors [43].

Although these adhesives can be used with either the total-etch or self-etch technique, there is a consensus in the literature that selective enamel conditioning improves adhesion strength. However, the favorable effect of acid etching on enamel before applying these adhesives to dentin is still debated in the literature and may vary depending on the brand or acidity intensity of the functional monomer. Because these adhesives contain primer and bond in one bottle, they have a high amount of hydrophilic monomers and solvents, making it necessary to apply an air jet to evaporate these solvents for at least 15 s [42,43].

Although significant advancements have been achieved with more recent adhesive systems, the most referenced adhesives appear to have remained unchanged for years. It is essential to recognize that this outcome may be attributed to the extensive number of studies conducted on these adhesives, the lack of sufficient research on competing adhesives, or even the techniques employed in these studies. Based on the available scientific literature, it can still be stated that for the total-etch technique, the most recommended adhesive remains OptiBond FL (Kerr) (Orange, CA, USA), while for the self-etch technique (with selective enamel etching), the most recommended adhesive is Clearfil SE Bond (Kuraray Noritake) (Okayama, Japan) [44].

### 4.1. Referenced Technique for Total Etch (Optibond FL)

**Figure 8:**
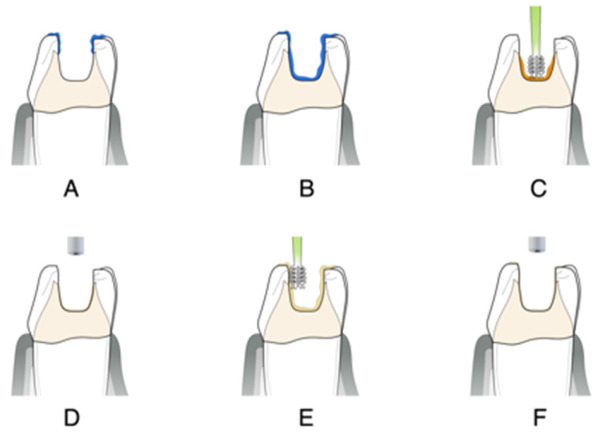
Illustration of the total-etch technique. (**A**) Selective enamel etching. (**B**) Total etch. (**C**) Primer application. (**D**) Solvent evaporation. (**E**) Bond application. (**F**) Excess removal.

After proper isolation of the operatory field, the substrate that will receive the bonding protocol should follow the mechanical cleaning and roughening protocol or follow the APA protocol [1,2,3,4,5], as follows:

- Apply selective enamel etching with 30% to 40% phosphoric acid gel for a period of 15 s. After this period, apply the acid to the dentin surface for 10–15 s (maximum) * (Figure 8A,B).
- Thoroughly water rinse for 10–15 s and air dry the enamel surface. Air dry the dentin after to remove visible water.
- Actively rub the primer on the dentin surface for at least 15 s *. Extra application or extra time with rubbing the primer is always welcome (Figure 8C).
- Gently air-dry the primer for at least 15 s to evaporate solvents * (Figure 8D).
- Apply the “bond” on all etched surfaces, ideally with a fiber-free micro brush (Figure 8E).
- With gentle air blowing and suction, remove the excess while uniformly spreading the adhesive resin (Figure 8F).
- Photopolymerize for 40 s.

* Only if dentin is exposed.

### 4.2. Referenced Technique for Self-Etch (Clearfil SE)

**Figure 9:**
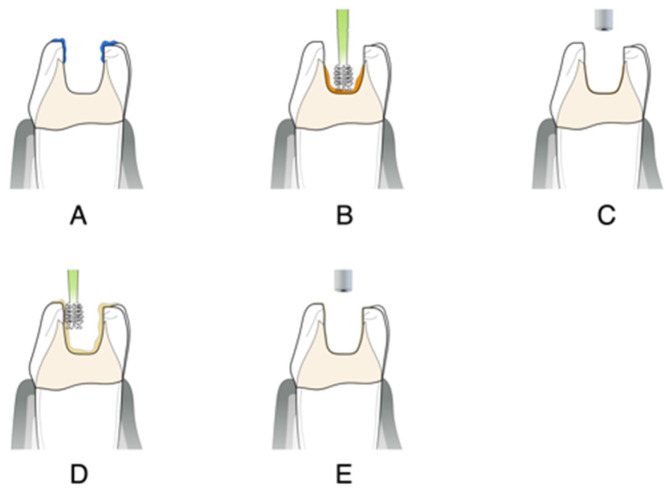
Illustration of the self-etch technique. (**A**) Selective enamel etching. (**B**) Primer application. (**C**) Solvent evaporation. (**D**) Bond application. (**E**) Excess removal.

After proper isolation of the operatory field, the substrate that will receive the bonding protocol should follow the mechanical cleaning and roughening protocol or follow the APA protocol [1,2,3,4,5], as follows:

- Apply selective enamel etching with 30% to 40% phosphoric acid gel for 15–30 s (Figure 9A).
- Thoroughly water rinse for 10–15 s and air dry.
- Actively rub the primer on the dentin surface for at least 15 s *. Extra application or extra time with rubbing the primer is always welcome (Figure 9B).
- Gently air dry the primer for at least 15 s to evaporate solvents * (Figure 9C).
- Apply the “bond” on all etched surfaces, ideally with a fiber-free micro brush (Figure 9D).
- With gentle air blowing and suction, remove the excess while uniformly spread the adhesive resin (Figure 9E).
- Photopolymerize for 40 s.

* Only if dentin is exposed.

Tip: Caution must be exercised when cementing indirect restorations, as a thick layer of photoactivated adhesive may hinder the proper fit of the restoration. To avoid this problem, air thinning should be performed before polymerization, or polymerization should be carried out after positioning the restoration with the chosen cement.

### 4.3. Different Approaches to Different Clinical Situations

Small changes in clinical approach can be made depending on the type of restoration being performed. The time and method for achieving better adhesion can differ between direct and indirect restorations. As mentioned before in this article, the best time to make an adhesive procedure on dentin is at the moment of preparation. This can be an advantage of direct or chairside restorations, since the adhesive procedure and the restoration will be performed consecutively. For indirect restorations (onlays, inlays, overlays, and crowns), the final restoration has to be made in the lab; hence, there will be a provisional restoration to cover the prepared surface, and the final adhesion is only made when the indirect restoration arrives from the lab. This is a downside for indirect restorations, since during the provisional phase, the exposed dentin can be contaminated, and if not treated properly upon cementation, bond strength may be reduced drastically [17].

**Figure 10:**
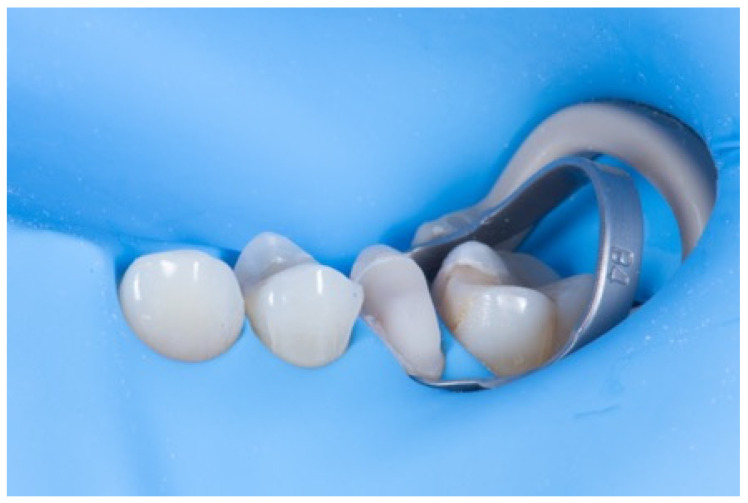
IDS performed on the pre-molar for indirect restoration.

To mitigate these problems, immediate dentin sealing (IDS) may be performed to secure the bond strength at the moment of preparation for indirect restorations. The IDS goal is to make an adhesive procedure right after the dentin preparation and seal the dentin with a filled bonding agent or a flowable composite [17,18]. This will ensure that the best adhesion on dentin is performed at the correct time, preventing dentin contamination (Figure 10).

**Figure 11:**
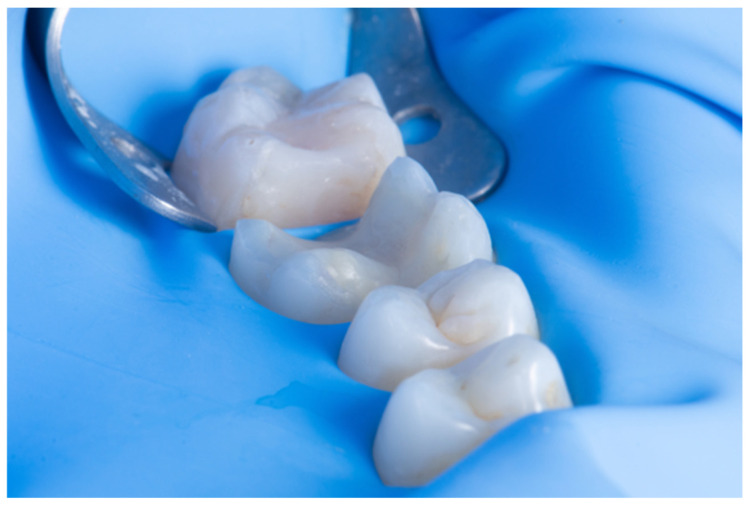
IDS and preparation under rubber dam.

**Figure 12:**
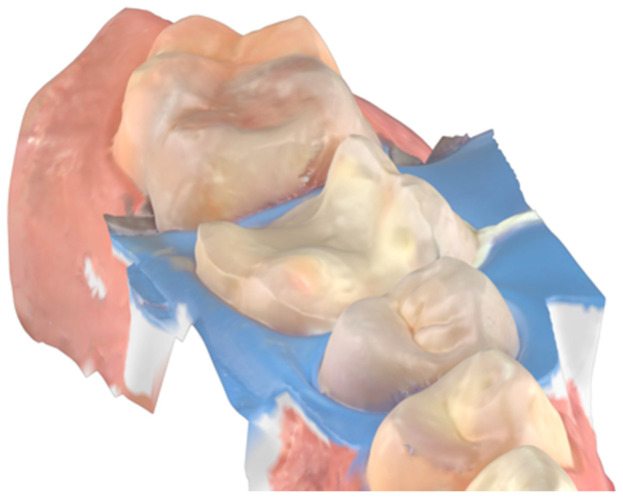
Intra-oral scan under rubber dam.

**Figure 13:**
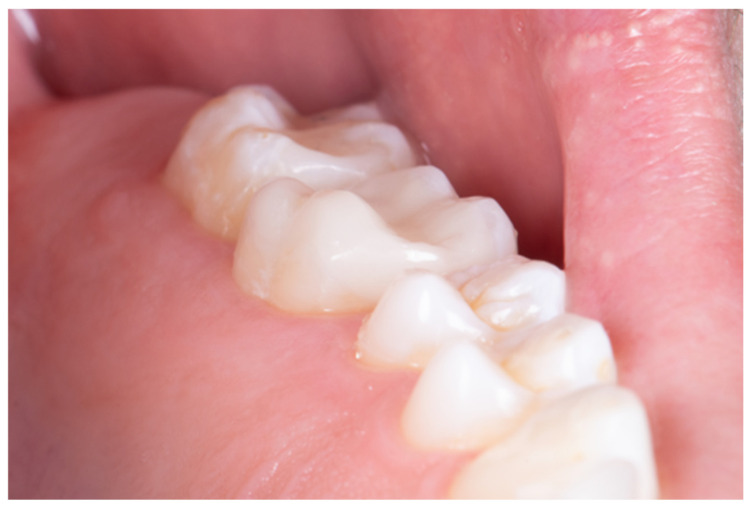
Final restoration.

IDS is an excellent procedure for indirect restorations on the posterior tooth or with dental crowns, and with the advantage of intra-oral scanners, the digital impression can be made right after IDS while the tooth is still under a rubber dam. This technique, described by Victor Clavijo [35,36], aims to reduce clinical time while ensuring the best operative site for digital impressions. The main challenges of using intra-oral scanners include the presence of fluids that can cause distortions in the scan and difficulties with tissue retraction, as the scanner can only copy what it can see. Rubber dam isolation provides an operative site with optimal tissue retraction and no fluids, allowing for the digital impression to perform effectively. An effective approach for indirect restorations involves rubber dam isolation, followed by the removal of carious lesions, unsatisfactory restorative materials, or provisionals after endodontic treatment. This is then followed by immediate dentin sealing (IDS), preparing for the desired restoration thickness, and, finally, the intra-oral scan (Figure 11, Figure 12 and Figure 13) [35,36].

The primary drawback of the IDS technique occurs when preparation cannot be performed under rubber dam isolation. Laminate veneers are an example where IDS may be ineffective. Due to the necessity of performing preparation with a mock-up in place [37], controlling dental preparation using silicone guides, and consistently viewing the gingival margins, it is impractical to carry out preparation under a rubber dam and follow it with IDS. One alternative might be to complete the dental preparation and then perform rubber dam isolation. The main issue with this alternative is that the preparation for laminate veneers is precisely calculated to avoid unnecessary removal of dental tissue; therefore, there would be no space to perform IDS. Consequently, the clinician would need to deepen the preparation where dentin is present to create space for IDS without compromising the restoration thickness.

Immediate dentin sealing (IDS) has been increasingly supported by the contemporary literature as an effective strategy for preserving dentin bond strength in indirect restorations. Clinical and in vitro studies demonstrate that sealing freshly cut dentin reduces bacterial penetration, minimizes postoperative sensitivity, and improves resin–dentin bond stability at the time of final cementation [17,18,37].

The integration of IDS with digital workflows, including intra-oral scanning under rubber dam isolation, represents a clinically relevant advancement. Emerging evidence suggests that optimal tissue retraction and moisture control achieved by rubber dam isolation enhance scan accuracy and restorative fit [35,36]. Nonetheless, limitations persist in scenarios where rubber dam placement is incompatible with preparation design, such as minimally invasive laminate veneers [37].

## 5. Conclusions

This review has limitations due to its narrative design. Although a structured search strategy and careful selection of references were used, the heterogeneity between laboratory methodologies and clinical protocols limits direct comparability between studies, especially regarding the heterogeneity of sources and the absence of quantitative analysis. The databases searched were PubMed, Scopus, and Web of Science.

Strict adherence to evidence-based clinical protocols is just as important for predictable dental adhesion as material selection. Integrating absolute isolation, suitable substrate preparation, and proven adhesive techniques results in long-term success. The durability of both direct and indirect restorations is eventually improved by multi-step techniques, which continue to show higher clinical reliability even though simplified systems provide procedural efficiency.

## Data Availability

No new data were created or analyzed in this study.
